# Correlation between Diabetes Mellitus and Knee Osteoarthritis: A Dry-To-Wet Lab Approach

**DOI:** 10.3390/ijms19103021

**Published:** 2018-10-03

**Authors:** Navneet Kumar Dubey, Dina Nur Anggraini Ningrum, Rajni Dubey, Yue-Hua Deng, Yu-Chuan Li, Peter D. Wang, Joseph R. Wang, Shabbir Syed-Abdul, Win-Ping Deng

**Affiliations:** 1 Graduate Institute of Biomedical Materials and Tissue Engineering, College of Biomedical Engineering, Taipei Medical University, Taipei 110, Taiwan; bioengineer.nkd@gmail.com; 2 Stem Cell Research Center, Taipei Medical University, Taipei 110, Taiwan; yuehuahua828@gmail.com; 3 Graduate Institute of Biomedical Informatics, College of Medical Science and Technology, Taipei Medical University, Taipei 110, Taiwan; dinanan@mail.unnes.ac.id (D.N.A.N.); jack@tmu.edu.tw (Y.-C.L.); 4 International Center for Health Information Technology, College of Medical Science and Technology, Taipei Medical University, Taipei 110, Taiwan; 5 Public Health Department, Universitas Negeri Semarang, Semarang City 50229, Indonesia; 6 Graduate Institute Food Science and Technology, National Taiwan University, Taipei 106, Taiwan; rajni.nitrkl@gmail.com; 7 Department of Life Science, Fu Jen Catholic University, New Taipei City 242, Taiwan; 8 Department of Dentistry, Taipei Medical University Hospital, Taipei 110, Taiwan; dpw1@tmu.edu.tw; 9 School of Dentistry, College of Oral Medicine, Taipei Medical University, Taipei 110, Taiwan; 10 Department of Periodontics, College of Dental Medicine, Columbia University, New York 10032, USA; jrw2166@cumc.columbia.edu; 11 Department of Basic Medicine, Fu-Jen Catholic University, Taipei 242, Taiwan

**Keywords:** diabetes mellitus, knee osteoarthritis, odds ratio, risk, articular cartilage, proteoglycan

## Abstract

Recent years have witnessed an increased prevalence of knee osteoarthritis (KOA) among diabetes mellitus (DM) patients—conditions which might share common risk factors such as obesity and advanced aging. Therefore, we conducted dry-to-wet lab research approaches to assess the correlation of type 1 DM (T1DM) and type 2 DM (T2DM) with KOA among all age and genders of Taiwanese population. The strength of association (odds ratio: OR) was analyzed using a phenome-wide association study portal. Populations of 37,353 T1DM and 1,218,254 T2DM were included. We observed a significant association of KOA with T1DM (OR: 1.40 (1.33–1.47), *p*< 0.0001) and T2DM (OR: 2.75 (2.72–2.78), *p*< 0.0001). The association between T1DM and KOA among the obese (OR: 0.99 (0.54–1.67), *p* = 0.0477) was insignificant compared to the non-obese (OR: 1.40 (1.33–1.48), *p* < 0.0001). Interestingly, a higher association between T2DM and KOA among non-obese persons (OR: 2.75, (2.72–2.79), *p* < 0.0001) compared to the obese (OR: 1.71 (1.55–1.89), *p* < 0.0001) was noted. Further, histopathologic and Western blot studies of diabetic mice knee joints revealed enhanced carboxymethyl lysine (advanced glycation end product), matrix metalloproteinase-1, and reduced cartilage-specific proteins, including type II collagen (Col II), SOX9, and aggrecan (AGN), indicating deteriorated articular cartilage and proteoglycans. Results indicate that DM is strongly associated with KOA, and obesity may not be a confounding factor.

## 1. Introduction

Diabetes mellitus (DM) and knee osteoarthritis (KOA) often coexist and share various risk factors such as obesity and aging. DM is a metabolic syndrome characterized by increased blood glucose levels and primarily falls into two broad categories: type 1 DM (T1DM) and type 2 DM (T2DM). Being a noncommunicable disorder, DM has been identified to exhibit the third-highest rate of mortality worldwide [1] and has been projected to escalate from 6.4% in 2010 to 7.7% in 2030 [2]. Approximately 5% of individuals develop DM per year [3]. It is also assumed that T1DM and T2DM are mostly prevalent in younger and aging populations, respectively, while a few reports have indicated the occurrence of T1DM even in aged populations and T2DM in younger persons [4,5,6]. That notwithstanding, both types of DM exhibit hyperglycemia as a common outcome [7], which results in downstream complications associated with connective tissues [8], renal function [9], the cardiovascular system [10], and the nervous system [11]. However, KOA as an anticipated diabetic complication has received a much less attention, and only a few reports and viewpoints indicate its possibility [12,13].

Based on previous reports, both types of diabetic populations are increasing at a staggering rate [14], which could be a risk factor for initiation or progression of KOA. KOA is a progressive diarthrodial joint disorder which leads to deterioration of articular cartilage (AC) and subchondral bone along with synovial inflammation, thereby disrupting the normal biomechanics of the knee joint, causing pain, stiffness, swelling, progressive deformity, and disability [15,16,17]. Since both DM and KOA are metabolic syndromes [18,19], it is hypothesized that the detrimental effect the of hyperglycemic condition could alter the metabolism of normal articular cartilage and associated tissues.

As mentioned previously, obesity is another complex and multifactorial disorder which might be a risk factor for the development of both forms of DM [20]. It also participates in the development of KOA through biomechanical and systemic modes. The biomechanical mode may be explained on the basis of the overloading of weight-bearing knee joints, which accelerates wear and tear of articular cartilage and leads to pathogenesis of KOA [21,22]. Under the systemic mode, inflammation is caused by the elevated secretion of proinflammatory cytokines, which compromises the integrity of the cartilage extracellular matrix [23].

Further, sex and ethnic/racial factors also have an influence over prevalence of diabetes [24]. Therefore, considering all the aforementioned pathophysiologic factors, our dry-lab approach investigated the possible association of T1DM and T2DM with KOA among the Taiwanese population by employing a phenome-wide association study (PWAS) portal developed with population-wide observational e-claim data from the National Health Insurance Research Database (NHIRD), one of the largest databases for Taiwan [25]. This portal provides the estimates of count, co-occurrence, odds ratio (OR: strength of association), *p* value, and 95% confidence interval (CI) while determining the association between the two disorders. So, the PWAS is an effective tool for comprehensively utilizing the information contained in electronic health records to understand the complex associations between these diseases. As a result, PWAS is helpful in generating testable hypotheses for its validation through experimental results. Therefore, in our study, the anticipated association outcomes of PWAS were corroborated through wet-lab experimentations. Specifically, we established experimental diabetes in non-obese mice by using streptozotocin (STZ), a diabetogenic agent [1], and their knee joints were harvested for histologic analysis through hematoxylin and eosin (for ultrastructure) and safranin O staining (for proteoglycans). Further, we examined the expression levels of knee-joint articular cartilage-specific proteins, such as type II collagen (Col II), SOX9, and aggrecan (AGN) to confirm cartilage degeneration. Taken together, we aimed to confirm the anticipated dry-lab outcomes of association between DM and KOA through wet-lab experimentations.

## 2. Results

**Figure 1:**
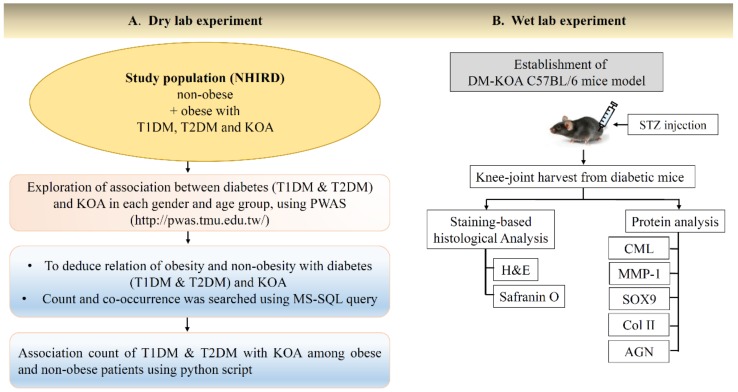
Schematic diagram of dry- and wet-lab experiments investigating the potential correlation between diabetes mellitus (DM) and knee osteoarthritis (KOA). (**A**) Selection of study population from NHIRD, Taiwan for exploration of association between DM and KOA among obese and non-obese subjects. (**B**) Establishment of diabetic KOA in C57BL/6J mice. DM: Diabetes mellitus, T1DM: Type 1 diabetes mellitus, T2DM: Type 2 diabetes mellitus, KOA: Knee-osteoarthritis, NHIRD: National Health Insurance Research Database, PWAS: Phenome-wide association study, MS-SQL: Microsoft Structured Query Language, STZ: Streptozotocin, H&E: Hematoxylin and eosin, CML: Carboxymethyl lysine, MMP-1: Matrix-metalloproteinase-1, Col II: Type II collagen, AGN: Aggrecan.

Our study comprised a combined dry- and wet-lab approach for deducing the correlation between DM and KOA, as shown in Figure 1.

### 2.1. Association between T1DM and KOA

**Figure 2:**
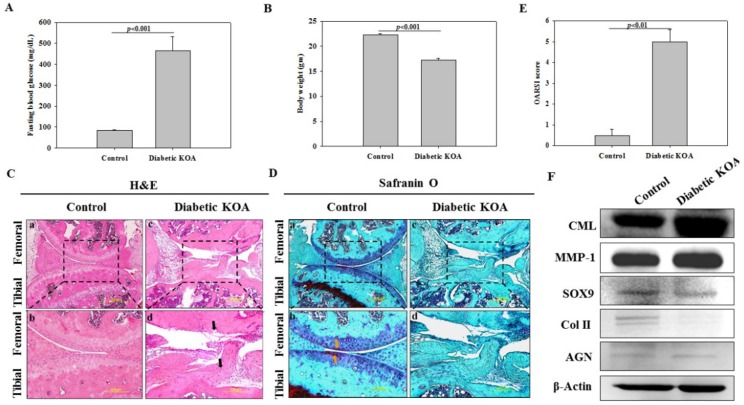
Characterization of KOA in diabetic mice after 4 weeks of STZ administration. Comparison of (**A**) blood glucose levels and (**B**) body weight. (**C**) H&E, and (**D**) safranin O staining for assessment of structure and distribution of red-colored proteoglycans (indicated by yellow arrows), respectively. Bar: 500 μm (lower magnification, 10×), 200 μm (higher magnification, 20×). (**E**) OARSI grade for assessing severity of articular cartilage degradation. Data are shown as mean ± SEM (Control, *n* = 5; Diabetic KOA, *n* = 5). (**F**) Western blot analysis of carboxymethyl lysine (CML), an advanced glycation end product; matrix-metalloproteinase-1 (MMP-1); and cartilage-specific proteins, including SOX9, type II collagen (Col II), and aggrecan (AGN) among control and diabetic KOA groups.

Obesity is a highly significant risk factor associated with the development of DM as well as KOA [26,27]. Hence, the co-occurrence and the OR demonstrating the association between T1DM and KOA among obese and non-obese subjects of both genders and all age groups with an interval of 10 years were computed (Table 1) using a PWAS derived from Taiwan’s NHIRD (Figure 2). The results of the PWAS were considered significant if the co-occurrence was more than 50; otherwise, it was assumed as bias such as miscoding. Thereafter, the associations among the comorbidities for the specific age group were analyzed. Interestingly, the highest risk of KOA was observed among adjusted non-obese females (OR: 1.44, CI: 1.14–1.81) and unadjusted obese males (OR: 1.46, CI: 1.17–1.82) in the age group of 80–89 and 50–59, respectively. Further, significant associations were also revealed in adjusted non-obese females in the age groups of 50–59 (OR: 1.30, CI: 1.12–1.50), 60–69 (OR: 1.23, CI: 1.12–1.35), and 80–89 (OR: 1.44, CI: 1.14–1.81). Similarly, adjusted non-obese males also showed a significant association in the age groups of 50–59 (OR: 1.45, CI: 1.16–1.81), 60–69 (OR: 1.45, CI: 1.26–1.68), and 70–79 (OR: 1.30, CI: 1.13–1.50), respectively. No significant association was found among obese females as well as males.

Notably, the calculated overall strength of association among non-obese subjects (OR: 1.40, CI: 1.33–1.48) was higher than that of obese subjects (OR: 0.99, CI: 0.54–1.67), which indicates that obesity is not the confounding factor (Table 2).

### 2.2. Association between T2DM and KOA

**Figure 3:**
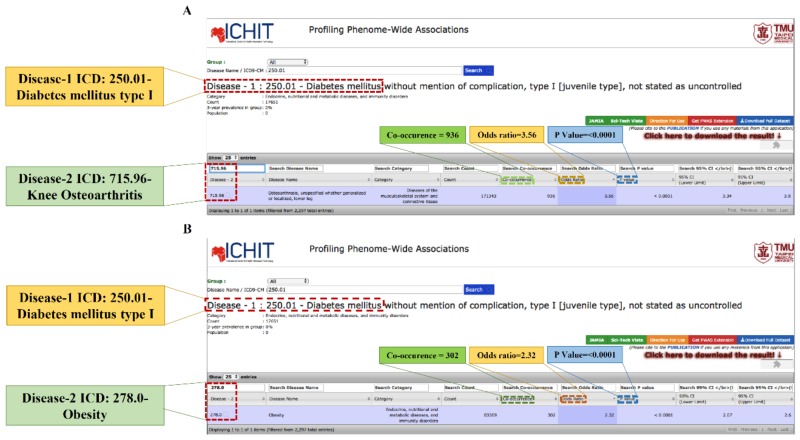
Screenshot of PWAS portal displaying the association between (**A**) T1DM and KOA and (**B**) T1DM and obesity. DM: Diabetes mellitus, T1DM: Type 1 diabetes mellitus, KOA: Knee osteoarthritis, and PWAS: Phenome-wide association study.

Table 3 shows the co-occurrence and the OR, which demonstrates the association between T2DM and KOA among obese and non-obese persons using the PWAS derived from NHIRD (Figure 3). T2DM also demonstrated the highest risk of KOA among unadjusted obese females (OR: 2.77, CI: 2.46–3.12) and males (OR: 2.68, CI: 2.34–3.06) in age group of 30–39. Further, the significant associations among obese females were found only in the age groups of 60–69 (OR: 1.41, CI: 1.15–1.74), whereas no significant association was found among obese males. Therefore, due to the higher association between DM and KOA among non-obese subjects, we further conducted wet-lab experiments to verify our observed outcomes in streptozotocin-derived, diabetes-induced KOA in non-obese C57BL/6J mice.

In agreement with the T1DM group, the overall strength of association among non-obese subjects (OR: 2.75, CI: 2.72–2.79) was again found to be higher compared to obese subjects (OR: 1.71, CI: 1.55–1.89) (Table 4).

### 2.3. Characterization of DM

Increased blood glucose level and decreased body weight are the hallmarks of DM [24]. At 4 weeks, compared to control, fasting blood glucose (FBG) was significantly increased in the diabetic KOA group, which reached an average of 470 mg/dL (Figure 2A), in addition to decreased body weight (Figure 2B). This clearly indicated the phenotype of diabetes in STZ-injected mice.

### 2.4. Staining-Based Assessment of KOA-Like Phenotype in Diabetic Mice

Articular cartilage (AC) is a primary osteochondral functional unit of the knee joint and its intactness is a pre-requisite for low-friction articulation, pressure dispersion, and preserving functional integrity [28]. Hence, to assess KOA-like pathology in diabetic mice, designated as diabetic KOA, H&E staining was conducted. We found that the control group revealed normal-appearing articular cartilage (Figure 2C-a) and the uppermost superficial acellular layer containing dense collagen fibres was intact and smooth. Many round, smaller, and flattened (spindle-shaped) chondrocytes housed in lacuna were present and were predominantly parallel to the articular surface (Figure 2C-b). Moreover, isogenous chondrocytes were surrounded by tangentially arranged collagenous fibres. The territorial and interterritorial matrices were well organized and more representative of healthy AC, whereas a huge deterioration of AC in the diabetic KOA group was evident (indicated by black arrowheads) with a complete loss of the superficial zone (Figure 2C-c). Extremely loosened plexuses of wavy collagen fibrils with an absolute loss of chondrocytes were observed with no zonation. Invagination extending vertically deeper from the articular surface to subchondral bone and fibrillation were also noticed (Figure 2C-d). Overall, the control group did not exhibit any sign of structural damage, while massive pathologic alterations were exhibited in diabetic KOA group.

Further, safranin O staining was conducted to determine the magnitude of aggrecan loss, which is a sulfated glycosaminoglycan (sGAG) and is a hallmark of cartilage degradation. In the control group, AC demonstrated smoother femoral and tibial surfaces and the absence of fibrillation; in addition, the extracellular matrix appeared evenly stained red (indicated by yellow arrowheads) (Figure 2D-a). Furthermore, the intensive red color in territorial matrices indicated proteoglycan-rich extracellular regions (Figure 2D-b). On the other hand, the diabetic KOA group exhibited the absence of cellularity and almost no staining in femoral regions, while feeble staining was found in the tibial region (Figure 2D-c), suggesting a massive loss of sGAG and thinned GAG-rich zones (Figure 2D-d) that demonstrated the osteoarthritic phenotype. Further, based on the above histological characteristics, the severity of KOA was graded by using the Osteoarthritis Research Society International Initiative (OARSI) score [29], which was significantly higher in the diabetic KOA group compared to control (Figure 2E).

### 2.5. Determination of Accumulated Advanced Glycation End Product (AGE) and Inflammatory Degradative Enzyme

We further investigated whether high-glucose-induced carboxymethyl lysine (CML), an AGE, was enhanced in the knee joint. Western blot analysis revealed a higher expression of CML protein in the diabetic KOA group compared to the control (Figure 2F, CML panel), indicating accelerated accumulation of glycated proteins. Matrix metalloproteinase (MMPs) are well-known inflammatory mediators of matrix degradation, and their activity by virtue of the cleavage of matrix substrates has been implicated in OA development [30]. Our results also showed increased levels of MMP-1 in the diabetic KOA group compared to the control (Figure 2F, MMP-1 panel), implying an enhanced magnitude of inflammation.

### 2.6. Expression of Cartilage-Specific Proteins

To investigate the effect of diabetes on the chondrogenic phenotype, Western blotting was further performed to determine expression patterns of SOX9, Col II, and AGN, which is a natural cartilaginous extracellular matrix component of AC (Figure 2F, SOX9, Col II, and AGN panels, respectively). The results revealed that expression of SOX9, an essential transcription factor for differentiated chondrogenic phenotype, was significantly reduced in the diabetic KOA group compared to the control group. Moreover, Col II and AGN, key cartilaginous matrix components, were highly diminished in the diabetic KOA group compared with the control, which is a major indication towards development of OA.

## 3. Discussion

Owing to disparities existing in the prevalence of diabetes due to racial, ethnic, and geographical attributes, we conducted a large-scale, population-based study using a PWAS derived from Taiwan’s NHIRD to deduce the possible association of both types of diabetes (T1DM and T2DM) with KOA among all ages and genders. Importantly, the uniqueness of the PWAS lies in the fact that this comprehensive database provides an insight into the understanding of disease pathogeneses and their associations. In a recent literature review and meta-analysis by Louati et al., the risk of KOA in DM patients was reported with an OR of 1.46 [31], whereas the association found in our study is much higher in T2DM subjects (OR: 2.75), which may be ascribed to different sample size, geographic location, and variations in candidate gene regions affecting diabetes within different ethnic groups [32]. Further, the higher prevalence of symptomatic KOA among Chinese women may also be a cause behind the high OR obtained in this study [33]. In agreement with prior studies reporting a higher risk of developing DM among Zuni Indian females than males [34], the estimated higher strength of association of both T1DM and T2DM with KOA in females is also evident in our study.

In the present study, the observed higher association in female patients might be attributed to various factors, including anatomical differences (lesser total tibial and patella cartilage volume) as well as genetic, hormonal issues, kinematic, and kinetic characteristics [35,36]. However, we found no data for the association below 30 years, indicating a very rare chance of incidence at this stage. It is known that both T2DM and KOA is more prevalent in people above 55 years [37,38], which was reflected by the high co-occurrence in our study. However, the high OR at the age group of 30–50 suggests that young adults are also susceptible and hence are at very high risk. Notably, the higher association between DM and KOA in non-obese patients in this study is contrary to previous reports showing obesity as a possible confounder [39,40]. The co-occurrence of less than 51 was found below the age of 30 years in T2DM, as the cases of occurrence of T2DM and KOA below 30 are very low and the frequency of having both disorders at this age is reduced or negligible.

It is well known that KOA is distinguished by the erosion of articular cartilage, which possesses poor self-healing capacity due to the limited regeneration in chondrocytes [41]. Our staining and Western blot results showing degraded cartilage and proteoglycans agree with osteoarthritic characteristics. Besides, while the exact mechanism involving DM and KOA is still debatable, there are few suggestive pathophysiological mechanisms for the development of OA in the diabetic microenvironment. One of them is hyperglycemia-induced accelerated synthesis of AGEs and their accumulation in normal articular cartilage matrix [42], which leads to an increase in oxidative stress [43]. These AGEs have been touted as one of the factors responsible for healing impairment and loss of elasticity [44]. Based on this assumption, our results demonstrated a higher accumulation of CML (AGE) in in the knee joints of diabetic mice that was similar to a study by Yu et al. demonstrating enhanced glycation in the kidneys of diabetic mice [45], which might exert elevated oxidative stress. Glycation is known to induce MMPs in the hyperglycemic microenvironment, which supports our data exhibiting a higher expression of MMP-1 in the knee joints [46] of diabetic mice, indicating an inflammatory phenotype. Further, the reduced expression of chondrocyte-specific proteins, including SOX9, Col II, and aggrecan, is the hallmark of OA pathophysiology [47] and also coincides with our results. In an interesting study, the loss of viscoelastic ligaments in the knee joints of diabetic rats further indicates the possibility of adverse changes in structural and functional characteristics leading to OA in a hyperglycemic microenvironment [48]. Based on the data, including strength of association (OR), histologic analysis, and Western blot studies, this is the first dry-to-wet approach study providing insight into the association between DM and KOA. This preliminary study has many strengths. The PWAS used in this study provides disease associations among all age groups and in both genders. The higher OR between DM and KOA obtained from PWAS validated the hypothesis regarding the association between these comorbidities. On the other hand, H&E and safranin O staining clearly demonstrated massive deterioration of the knee-joint ultrastructure and proteoglycans in the articular cartilage matrix, respectively. Despite these strengths, our study also has several limitations. The PWAS can only be used for assessing associations between two disorders at a time. Therefore, multiple disorder associations could not be determined. This observational database could be employed to identify potential associations which warrant further consideration; however, it should not imply causality. Furthermore, though histologic and Western blot studies revealed degenerated articular cartilage and proteoglycans, the underlying definitive mechanism remains to be elucidated.

## 4. Materials and Methods

### 4.1. Dry-Lab Experiments

#### Data Source

We used a PWAS portal (available online: http://pwas.tmu.edu.tw/index.php) developed by employing patient phenotypes information from one of the largest administrative healthcare databases in the world to evaluate disease-wide associations [25]. Taiwan’s National Health Insurance (NHI) is reliable and covers 97% of inhabitants and offers extensive hospitalization and ambulatory care [49]. Patients with DM and KOA were identified through claimed visiting files of NHIRD. Everyone in Taiwan has a unique personal identification number (PIN), and to maintain patients’ privacy, the identity data were cryptographically scrambled by the NHIRD. Syed-Abdul et al. inspected 782 million outpatient visits that were observed in the entire Taiwanese population of over 22 million individuals for a period of 3 consecutive years (2000–2002) acquired from NHIRD [25]. The PWAS assumes that two diseases co-occurred if and only if they were recorded for the same individual within the 36-month observation period despite the number of times a phenotype was noted for the same individual. In this study, a total of 37,353 T1DM (1584 KOA + 35,769 non-KOA + 15 obese + 1569 non-obese) and 1,218,254 T2DM (41,325 KOA + 1,176,929 non-KOA + 667 obese + 40,658 non-obese) persons were included. From the PWAS webpage, the association between T1DM and KOA was determined using International Classification of Diseases, 9th revision, clinical modification (ICD-9-CM) diagnostic codes as 250. * 1, 250. * 3 (for T1DM) and KOA (715.96) (Figure 3). The asterisk (*) mark in diagnostic codes of T1DM denote values from 0–9.

**Figure 4:**
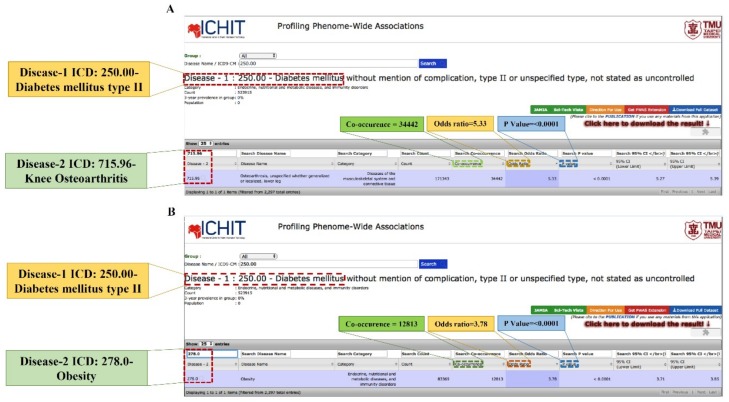
Screenshot of the PWAS portal displaying the association between (**A**) T2DM and KOA and (**B**) T2DM and obesity. DM: Diabetes mellitus, T2DM: Type 2 diabetes mellitus, KOA: Knee osteoarthritis, and PWAS: Phenome-wide association study.

Further, the association between T2DM and KOA was assessed using ICD-9-CM codes as 250. * 0, 250. * 2 (T2DM) and KOA (715.96) (Figure 4). The asterisk (*) mark in diagnostic codes of T2DM denote values from 0–9.

This webpage provides all essential values such as the count, co-occurrence, and OR with 95% CI for T1DM and T2DM with KOA among all age groups and genders. Using the PWAS portal, we retrieved OR values that indicated the strength of association between these comorbidities, which was complemented by *p*-values (*p* < 0.0001) and 95% CI limits based on hypergeometric tests [25]. OR higher than 1 and *p* ≤ 0.05 was considered as an enhanced risk of KOA among T1DM and T2DM patients. After exploring the count and co-occurrence, the patients were divided into obese (278 *) and non-obese (without 278) groups using Microsoft Structured Query Language (MS-SQL) queries. Eventually, the association count of T1DM and T2DM with KOA among obese and non-obese subjects was conducted using a Python script.

### 4.2. Wet-Lab Experiments

#### 4.2.1. Induction of Experimental Diabetes in Mice

Seven-week-old C57BL/6J male mice were purchased from the National Laboratory Animal Center, Taipei, Taiwan and were kept at the Laboratory Animal Center, Taipei Medical University (TMU). Animals were acclimatized to the laboratory conditions for 2 weeks and were fed with normal chow (LabDiet 5010). The DM in mice was established through a single intraperitoneal injection of 200 mg/kg streptozotocin (STZ: S0130, Sigma-Aldrich, St. Louis, MO, USA) freshly prepared in citrate buffer (pH 4.5).

#### 4.2.2. Monitoring of Blood Glucose Level

To confirm the presence of DM, blood samples were obtained from the tail vein of STZ-administered mice, and fasting blood glucose (FBG) was measured by glucose oxidase strips (Easytouch, Taipei, Taiwan). After 4 weeks of STZ treatment, the mice were considered diabetic when their blood glucose level exceeded 250 mg/dL [23] and were then selected for further experiments, including histopathologic and molecular level studies associated with KOA. These mice were assigned as the diabetic KOA group (*n* = 5), while the non-STZ-treated group was considered as the control group (*n* = 5).

#### 4.2.3. Histologic Analysis

After 4 weeks of STZ treatment, the diabetic KOA group and control mice were sacrificed (*n* = 5 animals/group). Knee joints were harvested and fixed in neutral formalin for 2 days and decalcified using a rapid decalcifier (Nihon Shiyaku Industries Ltd., Osaka, Japan) for further sectioning. After decalcification, the samples were embedded in paraffin and sectioned at 5-μm thickness along the sagittal plane. Slides were stained with H&E for assessment of the tissue architecture of articular cartilage. Safranin O staining and fast green staining (Sigma-Aldrich) were performed to analyze the distribution pattern of proteoglycans.

#### 4.2.4. Western Blot Analysis

Following 4 weeks of STZ treatment, mice from control and diabetic KOA groups were sacrificed. For protein extraction, knee-joint tissues were isolated, pulverized, and suspended in 100 μl 1X RIPA lysis buffer (Cat. No. 20–188, Millipore, Temecula, CA, USA), protease inhibitor cocktail set III, EDTA-Free (Cat. No. 539134, Millipore, USA), phosphatase inhibitor (Na_3_VO_4_), and sonicated. After 20 min of incubation on ice, samples were then centrifuged at 12,000 rpm for 40 min at 4 °C and the supernatant was collected for quantification. The protein samples were resolved on 10% SDS-PAGE gel and transferred to a PVDF (Polyvinylidene Fluoride, Amersham Hybond-P, GE Healthcare, Little Chalfont, UK) membrane. After blocking, membranes were incubated for 1 h at room temperature in PBST buffer with the CML, MMP-1, SOX9, Col II (Abcam, Cambridge, MA, USA), and aggrecan antibody (Millipore), respectively. This was followed by four washings for 10 min each at room temperature. Horseradish peroxidase-conjugated anti-rabbit IgG secondary antibody (Jackson ImmunoResearch, West Grove, PA, USA) was diluted in PBST (PBS with 0.05% Tween-20, pH 7.0) and incubated with blots for 1 h at room temperature. Immunoreactivity expressions were measured by developing blots using ECL plus-kit (Amersham Pharmacia, Piscataway, NJ, USA). Blots were visualized by UVP BioSpectrum^®^ imaging system.

## 5. Ethical Statement

For the dry-lab approach, as per the regulations of National Health Research Institutes (NHRI), informed consent was not required because the patients’ personal information had been decoded and scrambled. During wet-lab experiments, all the animal care and use protocols were in accordance with guidelines and received prior approval from the Institutional Animal Care and Use Committee, Taipei Medical University, Taiwan (no. LAC-2016-001, 30 September 2016).

## 6. Statistical Analysis

For the dry-lab experiments, the PWAS results are expressed in the terms of co-occurrence, odds ratio with confidence interval (95%), and *p*-value. Statistical significance was determined using Fisher’s exact test (*p*-value < 0.05). For the wet-lab experiments, results are expressed as mean ± standard error of mean (SEM). Data were analyzed using Student's *t*-test and a *p*-value < 0.05 was considered statistically significant.

## 7. Conclusions

This is the first dry-to-wet-lab-based study to provide significant evidence for the correlation between DM and KOA. Specifically, the PWAS that resulted in a higher strength of association (OR) between DM and KOA was confirmed in non-obese diabetic mice (high blood-glucose level), showing depleted articular cartilage and proteoglycans (indicator of KOA). Our results also imply that DM is strongly associated with KOA, and obesity may not be a confounding factor. However, further extensive studies including mechanistic insight and clinical parameters are needed to confirm the correlation between these pathologies.

## Figures and Tables

**Table 1:** The co-occurrence and the OR for the association between T1DM and KOA observed among various age groups of both genders in the Taiwanese population. Co.: co-occurrence, OR: odds ratio, n/a = not available. * and ** indicate *p*-value less than 0.05 and 0.0001, respectively.

Age Group (Years)	Female						Male					
	Unadjusted		Adjusted Obese		Adjusted Non-Obese		Unadjusted		Adjusted Obese		Adjusted Non-Obese	
	Co.	OR (CI 95%)	Co.	OR (CI 95%)	Co.	OR (CI 95%)	Co.	OR (CI 95%)	Co.	OR (CI 95%)	Co.	OR (CI 95%)
50–59	191	1.28 * (1.11–1.48)	2	0.46 (0.11–1.89)	189	1.30 ** (1.12–1.50)	80	1.46 * (1.17–1.82)	1	1.65 (0.22–12.26)	79	1.45 * (1.16–1.81)
60–69	462	1.23 ** (1.12–1.36)	8	1.28 (0.61–2.68)	454	1.23 ** (1.12–1.35)	189	1.45 ** (1.25–1.68)	1	0.75 (0.10–5.57)	188	1.45 ** (1.26–1.68)
70–79	357	1.07 (0.96–1.19)	2	0.64 (0.15–2.72)	355	1.07 (0.96–1.19)	206	1.31 ** (1.14–1.50)	1	2.29 (0.28–18.70)	205	1.30 ** (1.13–1.50)
80–89	76	1.43 * (1.13–1.80)	0	0 (n/a)	76	1.44 * (1.14–1.81)	23	0.74 (0.49–1.12)	0	0 (n/a)	23	0.74 (0.49–1.12)
Total	1086	1.32 ** (1.24–1.40)	12	0.93 (0.47–1.67)	1074	1.32 ** (1.24–1.40)	498	1.44 ** (1.31–1.58)	3	1.31 (0.26–4.06)	495	1.44 ** (1.31–1.58)

**Table 2:** Overall association between T1DM and KOA among obese and non-obese subjects. Co.: co-occurrence, OR: odds ratio. ** indicate *p*-value less than 0.0001.

Age Group (Years)	Male & Female								
	Unadjusted			Adjusted Obese			Adjusted Non-Obese		
	Co.	OR (CI 95%)	*p*-Value	Co.	OR (CI 95%)	*p*-Value	Co.	OR (CI 95%)	*p*-Value
All age groups	1584	1.40 ** (1.33–1.47)	<0.0001	15	0.99 (0.54–1.67)	0.0477	1569	1.40 ** (1.33–1.48)	<0.0001

**Table 3:** The co-occurrence and odds ratio for association of T2DM with KOA observed among various age groups of both genders in the Taiwanese population (adjusted among obese and non-obese). Co.: co-occurrence, OR: odds ratio, n/a = not available. * and ** indicate *p*-value less than 0.05 and 0.0001, respectively.

Age Group (Years)	Female						Male					
	Unadjusted		Adjusted Obese		Adjusted Non-Obese		Unadjusted		Adjusted Obese		Adjusted Non-Obese	
	Co.	OR (CI 95%)	Co.	OR (CI 95%)	Co.	OR (CI 95%)	Co.	OR (CI 95%)	Co.	OR (CI 95%)	Co.	OR (CI 95%)
30–39	298	2.77 ** (2.46–3.12)	21	1.40 (0.86–2.27)	277	2.74 ** (2.43–3.10)	230	2.68 ** (2.34–3.06)	9	1.56 (0.72–3.37)	221	2.65 ** (2.31–3.04)
40–49	1896	2.05 ** (1.95–2.15)	98	1.22 (0.96–1.54)	1798	2.03 ** (1.93–2.14)	997	1.90 ** (1.78–2.03)	15	1.02 (0.56–1.88)	982	1.90 ** (1.78–2.03)
50–59	5650	1.52 ** (1.47–1.56)	182	1.12 (0.92–1.35)	5468	1.51 ** (1.46–1.56)	2067	1.55 ** (1.47–1.62)	23	1.20 (0.70–2.07)	2044	1.54 ** (1.47–1.62)
60–69	10,602	1.3 1** (1.28–1.34)	199	1.41 * (1.15–1.74)	10,403	1.30 ** (1.27–1.34)	4113	1.44 ** (1.39–1.48)	38	1.03 (0.66–1.60)	4075	1.44 ** (1.39–1.49)
70–79	8091	1.28 ** (1.24-1.31)	53	1.03 (0.70–1.49)	8038	1.28 ** (1.24–1.31)	4944	1.43 ** (1.39-1.48)	24	1.68 (0.91–3.12)	4920	1.43 ** (1.38–1.48)
80–89	1498	1.44 ** (1.35–1.53)	5	1.37 (0.38–4.98)	1493	1.44 ** (1.35–1.53)	939	1.54 ** (1.43–1.65)	0	0 (n/a)	939	1.54 ** (1.43–1.66)
Total	28,035	2.76 ** (2.72–2.80)	558	1.80 ** (1.61–201)	27,477	2.77 ** (2.73–2.81)	13,290	2.63 ** (2.58–2.68)	109	1.62 ** (1.25–2.08)	13181	2.64 ** (2.58–2.69)

**Table 4:** Overall association between T2DM and KOA among obese and non-obese subjects. Co.: co-occurrence, OR: odds ratio. ** indicate *p*-value less than 0.0001.

Age Group (Years)	Male & Female								
	Unadjusted			Adjusted Obese			Adjusted Non-Obese		
	Co.	OR (CI 95%)	*p*-Value	Co.	OR (CI 95%)	*p*-Value	Co.	OR (CI 95%)	*p*-Value
All age group	41,325	2.75 ** (2.72–2.78)	<0.0001	667	1.71 ** (1.55–1.89)	<0.0001	40658	2.75 ** (2.72–2.79)	<0.0001

## Abbreviations

DM	Diabetes mellitus
KOA	Knee osteoarthritis
AGE	Advanced glycation end product
CML	Carboxymethyl lysine
NHIRD	National Health Insurance Research Database
PWAS	Phenome-wide association study
Col II	Type II collagen
AGN	Aggrecan
